# Antimicrobial activity of antidepressants on normal gut microbiota: Results of the *in vitro* study

**DOI:** 10.3389/fnbeh.2023.1132127

**Published:** 2023-03-22

**Authors:** Grigory Rukavishnikov, Lubov Leonova, Evgeny Kasyanov, Vadim Leonov, Nikholay Neznanov, Galina Mazo

**Affiliations:** ^1^Social Neuropsychiatry Department, Bekhterev National Medical Research Center for Psychiatry and Neurology, Saint-Petersburg, Russia; ^2^Department of Natural Sciences, Technology and Environmental Studies, Södertörn University, Stockholm, Sweden; ^3^Geriatric Psychiatry Department, Bekhterev National Medical Research Center for Psychiatry and Neurology, Saint-Petersburg, Russia; ^4^Psychiatry and Addictions Department, Pavlov First Saint-Petersburg State Medical University, Saint-Petersburg, Russia

**Keywords:** antidepressants, depression, antibacterial, microbiota-gut-brain axis, growth activity

## Abstract

Currently, there is little published data on the effects of antidepressants on normal gut microbiota and the consequences of such effects on treatment outcomes.

**The aim of the study:** was to evaluate the growth kinetics of normal human gut microorganisms with antidepressants most common in routine clinical practice.

**Materials and methods:** Research objects were species of microorganisms representing normal gut microbiota: *Staphylococcus aureus* ATCC 25923, *Escherichia coli* ATCC 25922, *Candida albicans* ATCC 24433, *Bifidobacterium* 791, *Enterococcus faecalis* ATCC 29212, *Lactobacillus rhamnosus* ATCC 53103. All microorganisms were cultivated in Schaedler broth (HiMedia) under aerobic/anaerobic conditions. The active substances of all studied antidepressants (fluvoxamine, fluoxetine, escitalopram, duloxetine, venlafaxine, mirtazapine) were extracted from ground preparations by dimethyl sulfoxide and centrifuged. Each solution of antidepressants was added to a Schaedler broth containing a certain microorganism’s strain and diluted to final concentrations—200 μg/ml, 500 μg/ml, and 700 μg/ml. For a quantitative assessment of the effect, the specific growth rates (μ, h-1) of microorganisms were calculated as the slope of the initial part of the growth curve in coordinates (lnA, t). To evaluate the antidepressant effects on representatives of the normal microbiota *in vitro*, the following parameters were chosen: specific growth rate and IC50.

**Results:** All antidepressants had an inhibitory effect on the growth of all studied microorganisms. Fluvoxamine and venlafaxine had the least effect on the growth activity of all studied microorganisms. Fluoxetine showed a pronounced effect on growth activity against *E. coli*, *E. feacalis*, *S. aureus*, and the least effect against *C. albicans*. Escitalopram had a greater effect on the growth rate of *E. coli*, *E. feacalis*, *B. bifidum*, *L. rhamnosus*, and *C. albicans*, which puts it among the leaders in terms of its effect on the growth activity of the microorganisms we studied. Mirtazapine, according to the results of our experiment, showed the greatest activity against *L. rhamnosus* and *C. albicans*.

**Conclusions:** Our results confirm the effects of antidepressants on the growth activity of the normal gut microbiota individual strains. Further study of the antimicrobial activity of antidepressants may become one of the new directions for optimizing the personalized therapy of patients with depression.

## 1. Introduction

The data from the preclinical studies showed that the microbiota and the whole microbiome both could play a significant role in neurodegenerative and neuropsychiatric disorders (e.g., depressive disorder, bipolar disorder, schizophrenia, autism, Parkinson’s disease; Kelly et al., 2016; Codagnone et al., 2019). The association between the microbiota and the central nervous system is based on the concept of a bidirectional microbiota-gut-brain axis (Osadchiy et al., 2019). However, it is still not clear whether the changes in the microbiome are causally related to mental disorders or their direct consequences.

The available experimental data showed that the gut microbiota is able to improve depressive symptoms, and the use of pro- and prebiotics can lead to a decrease in cortisol levels and attenuate depressive-like and anxiety behavior in mice (Messaoudi et al., 2011). Moreover, there is data on the microbiota effects on the levels of neurotransmitters involved in the mechanisms of antidepressants action (Stasi et al., 2019).

There are also publications confirming the antimicrobial effects of antidepressants (Munoz-Bellido et al., 2000; Ayaz et al., 2015; Karine de Sousa et al., 2018). Thus, the selective serotonin reuptake inhibitors (SSRIs) showed synergistic activity when combined with some antibiotics against several bacteria, shown by a decrease in MICs, that converts strains previously resistant to the category of sensitivity, and modify physiological aspects related to pathogenicity (Munoz-Bellido et al., 2000). Sertraline previously showed strong intrinsic antibacterial and antifungal activities and has augmented the antibacterial activities of antibiotics (Ayaz et al., 2015). The association of fluoxetine with gentamicin and erythromycin *P. aeruginosa* and *E. coli* presented synergistic effects, demonstrating that this drug can selectively modulate the activity of antibiotics of clinical use (Karine de Sousa et al., 2018). Although the effects of antidepressants on microorganisms have been studied for a long period of time, the available research is primarily focused on pathogenic strains and the synergistic activity of antidepressants and antibiotics (Munoz-Bellido et al., 2000; Ayaz et al., 2015; Karine de Sousa et al., 2018). The antibacterial effect of SSRIs (sertraline, fluoxetine, and paroxetine) has been described against gram-positive bacteria such as *Staphylococcus aureus* and *Enterococcus* (Kaatz et al., 2003; Sun et al., 2019). However, there is little published data on the effect of antidepressants on normal gut microbiota and the consequences of their antibacterial effect on treatment outcomes.

The study of the antidepressants’ effects on the gut microbiota will allow both to determine microorganism’s potential role in the tolerance and side effects of psychopharmacological drugs and to assess the feasibility of using microbiota-modeling tactics to optimize the therapeutic strategies for patients with depression.

The aim of the study was to evaluate the growth kinetics of normal human gut microorganisms with antidepressants most common in routine clinical practice.

## 2. Materials and methods

### 2.1. Strains and growth conditions

Experiments with microorganisms were performed on the basis of the Microbiology Department of the Khanty-Mansiysk State Medical Academy. We used standard methods of microorganism cultivation on solid and liquid nutrient media. Research objects were species of facultative anaerobe or aerotolerant anaerobe microorganisms representing minor normal microbiota. *Firmicutes: Staphylococcus aureus* ATCC 25923 (*S. aureus*), *Bifidobacterium bifidum* 791 (*B. bifidum*), *Enterococcus faecalis* ATCC 29212 (*E. faecalis*), *Lactobacillus rhamnosus* ATCC 53103 (*L. rhamnosus*); *Gracilicutes: Escherichia coli* ATCC 25922 (*E. coli*) and *Fungi: Candida albicans* ATCC 24433 (*C. albicans*). All microorganisms were cultivated in Schaedler broth (HiMedia) at 37°C under aerobic/anaerobic conditions with shaking for the amount of time required.

### 2.2. Drugs

To study the effects of antidepressants on microorganisms, we selected antidepressants from different pharmacological groups that are most often used in clinical practice for major depression treatment. SSRIs (fluvoxamine, fluoxetine, escitalopram), selective serotonin, and noradrenaline reuptake inhibitors (SNRIs—duloxetine, venlafaxine) and noradrenergic and specific serotonergic antidepressants (NaSSA—mirtazapine) were used in the study.

All antidepressant preparations were ground under aseptic conditions. The active substances from ground preparations were been extracting by dimethyl sulfoxide (DMSO) for 12 h at 25°C. To separate the active substance from the excipients, the solutions were centrifuged at 5,000 rpm for 30 min and the supernatant (active substance) was separated from the sediment (excipients). In order to control that active substance was tuned into DMSO we use the classical gravimetric method (Ballinger and Gershon, 2011). We weighted a substance before and after incubation with DMSO on an analytical balance (Sartorius) having a maximum weighing capacity between 60 g and 520 g after heating the sample (30–45°C) to constant mass.

### 2.3. The growth kinetics of microorganisms with antidepressants

Each solution of antidepressants in DMSO was added to a Schaedler broth containing a certain microorganism’s strain and diluted to final concentrations—200 μg/ml, 500 μg/ml, and 700 μg/ml. A mixture of microorganisms and preparations was dispensed into a 96-well plate. Microorganism cultures in Schaedler broth with DMSO without antidepressants were used as controls. Further, microorganisms were cultivated in a photometer (Multiscan FC) at 37°C, wavelength *λ* = 540 nm, and optical density of suspension (A, optic unit) measurements were performed every hour (20 min) during the cultivation period.

For a quantitative assessment of the effect of the preparations, the specific growth rates (μ, h-1) of microorganisms were calculated as the slope of the initial part of the growth curve in coordinates (lnA, t).

To evaluate the antidepressant effects on representatives of the normal microbiota *in vitro*, the following parameters were chosen: specific growth rate and IC_50_. The IC_50_ is the concentration of an antidepressant at which the microbial growth rate was reduced to 50% of its original value.

### 2.4. Statistical analysis

All experiments were performed thrice (*n* = 3) with similar results. The data were analyzed and graphically visualized using Rstudio and Windows (GraphPad Software, United States^1^). The inhibition concentration (IC50) of the specific growth rate was determined using GraphPad Prism version 6.00 for Windows (GraphPad Software, United States^1^). The data points that were used to calculate such parameters were those that produced linear regression with correlation coefficients (r2) higher than 0.94.

## 3. Results

The addition of antidepressants to the medium led to a decrease in the growth activity of all studied microorganisms.

**Figure 1:**
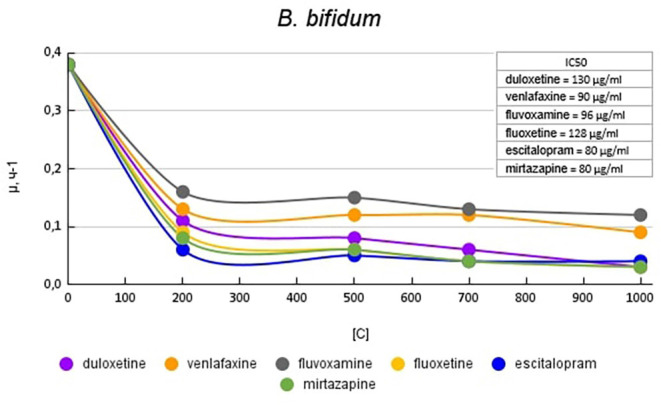
Specific growth rates of *B. bifidum* in the presence of antidepressants.

Figure 1 plots the growth rate of *B. bifidum* in the presence of antidepressants. The antidepressants escitalopram and mirtazapine had the greatest effect on growth activity. At a concentration of 200 μg/ml escitalopram and mirtazapine reduced the growth activity by 6.3 (84%) and 4.2 (76%) times compared with the control, respectively. Venlafaxine and fluvoxamine at a concentration of 200 μg/ml had the least effect on growth activity. The growth rate was 2.9 (65%) and 2.3 (56%) times compared with the control, respectively. When duloxetine and fluoxetine were added to the medium, a dose-dependent effect was observed. Duloxetine at a concentration of 200 μg/ml reduced the growth rate of *B. bifidum* by 3.4 (70%) times compared with the control. With a subsequent increase in the concentration of duloxetine, a consistent decrease in the specific growth rate of 4.7 (79%); 6.3 (84%), and 12.6 times compared to control was reported. Fluoxetine at a concentration of 200 μg/ml reduced the growth rate of *B. bifidum* by 4.2 (76%) times. With a subsequent increase in the concentration of fluoxetine, a decrease in the specific growth rate was observed by 6.3 (84%), 9.5 (89%), and 12.6 (92%) times compared with the control.

**Figure 2:**
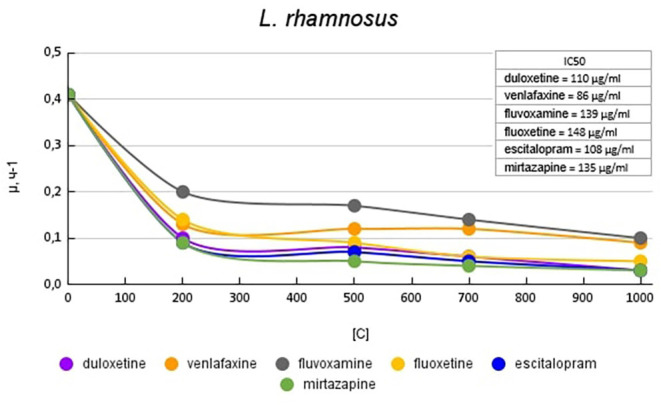
Specific growth rates of *L. rhamnosus* in the presence of antidepressants.

Figure 2 shows graphs of the specific growth rate of *L. rhamnosus* in the presence of antidepressants. The antidepressants escitalopram, mirtazapine, and duloxetine had the greatest effect on growth activity. At a concentration of 200 μg/ml, escitalopram, and mirtazapine reduced the growth activity by 4.5 (77%) times, and duloxetine at the same concentration by 4.1 (76%) times compared with the control. With a subsequent increase in the concentration of antidepressants in the medium, the growth rate changed less significantly. Fluvoxamine had the least effect on the growth activity of *L. rhamnosus*. At a concentration of 200 mg/ml, it reduced the growth rate by 2 (50%) times compared with the control.

**Figure 3:**
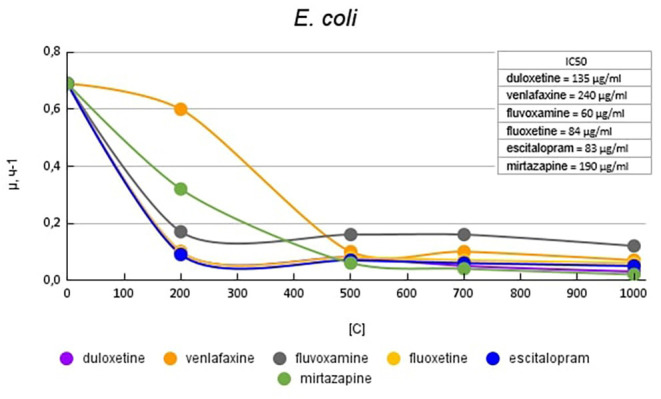
Specific growth rates of *E. coli* in the presence of antidepressants.

Figure 3 shows graphs of the specific growth rate of *E. coli* in the presence of antidepressants. The antidepressants duloxetine, fluoxetine, and escitalopram, had the greatest effect on growth activity. Duloxetine and fluoxetine at a concentration of 200 μg/ml reduced the growth rate by 6.9 (85%) times, and escitalopram at the same concentration reduced the growth rate by 7.6 (87%) times compared with the control. Venlafaxine and mirtazapine at a concentration of 200 μg/ml reduced the growth rate of *E. coli* to a lesser extent, by 1.2 (17%) and 2.2 (54%) times compared with the control, respectively. However, with a subsequent increase in the concentration of antidepressants, it led to a decrease in growth rate comparable to the above-described antidepressants. Fluvoxamine at a concentration of 200 mg/ml reduced the growth rate by 4.1 (76%) times compared with the control.

**Figure 4:**
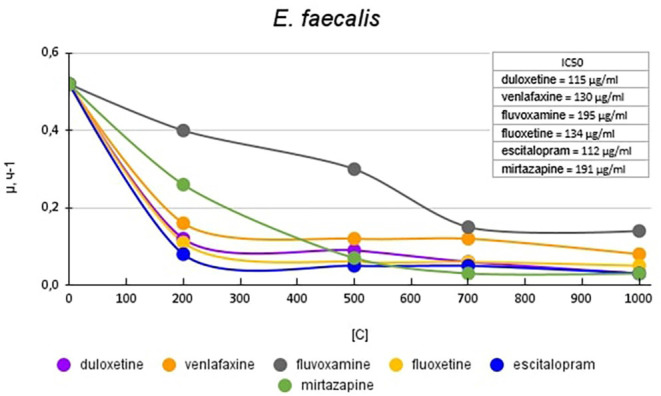
Specific growth rates of *E. faecalis* in the presence of antidepressants.

Figure 4 shows graphs of the specific growth rate of *E. faecalis* in the presence of antidepressants. The antidepressants duloxetine, fluoxetine, and escitalopram, had the greatest effect on growth activity. Duloxetine and fluoxetine at a concentration of 200 μg/ml reduced the growth rate by 4.1 (76%) and 4.7 (79%) times, and escitalopram at the same concentration reduced the growth rate by 6.5 (85%) times compared with the control. Mirtazapine at a concentration of 200 mg/ml reduced the growth rate of *E. faecalis* to a lesser extent, by 2 (50%) times compared to the control. However, with a subsequent increase in the concentration of antidepressants, it led to a decrease in growth rate comparable to the above-described antidepressants. Venlafaxine at a concentration of 200 mg/ml reduced the growth rate of *E. faecalis* by 3.3 (70%) times compared with the control. Fluvoxamine had the least effect on growth activity. At a concentration of 200 μg/ml, it reduced the growth rate by 1.3 (23%) times compared with the control.

**Figure 5:**
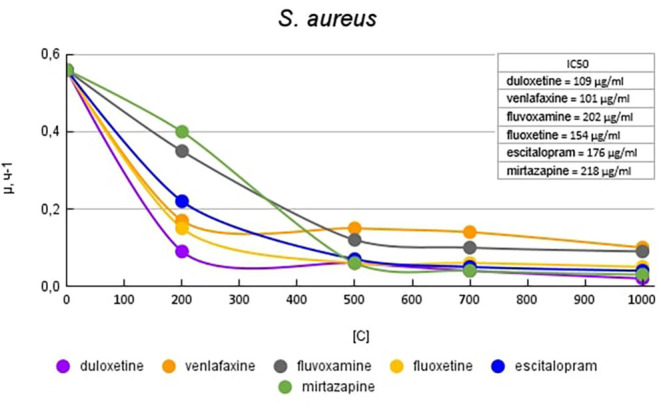
Specific growth rates of *S. aureus* in the presence of antidepressants.

Figure 5 shows graphs of the specific growth rate of *S. aureus* in the presence of antidepressants. Duloxetine had the greatest effect on growth activity; at a concentration of 200 μg/ml, it reduced the growth rate by 6.2 (84%) times compared with the control. Escitalopram and fluoxetine at a concentration of 200 mg/ml reduced the growth rate by 2.5 (60%) and 3.7 (73%) times compared with the control, respectively. However, a subsequent increase in the concentration of antidepressants led to a decrease in growth rate comparable to duloxetine. Mirtazapine at a concentration of 200 mg/ml reduced the growth rate by 1.4 (28%) times compared with the control. However, a subsequent increase in the concentration of antidepressants led to a decrease in growth rate comparable to duloxetine. Venlafaxine at a concentration of 200 mg/ml reduced the growth rate by 2.5 (60%) and 3.2 (69%) times compared with the control. Fluvoxamine at a concentration of 200 mg/ml reduced the growth rate by 1.6 (37%) times compared with the control. A subsequent increase in the concentration of antidepressants led to a decrease in growth rate comparable to that of venlafaxine.

**Figure 6:**
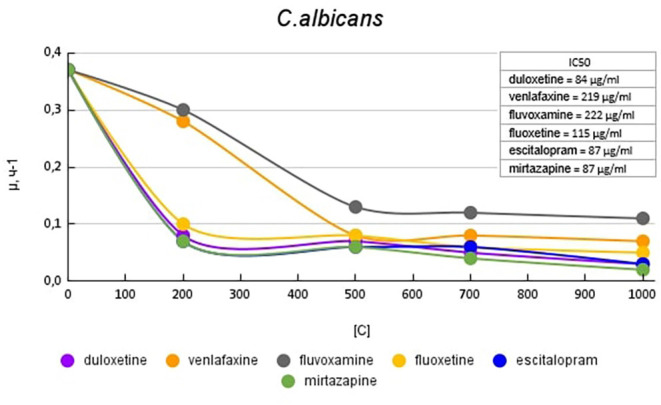
Specific growth rates of *C. albicans* in the presence of antidepressants.

Figure 6 shows plots of the specific growth rate of *C. albicans* in the presence of antidepressants. Duloxetine, mirtazapine, escitalopram, and fluoxetine had the greatest effect on growth activity. Mirtazapine and escitalopram at a concentration of 200 μg/ml reduced the growth rate by 5.3 (81%) times compared with the control. Fluoxetine and duloxetine at a concentration of 200 μg/ml reduced the growth rate by 3.7 (73%) and 4.6 (78%) times compared with the control. With a subsequent increase in the concentration of the antidepressant in the medium, the growth rate changed less significantly. Venlafaxine at a concentration of 200 μg/ml reduced the growth rate by 1.3 (23%) times compared with the control. However, with a subsequent increase in the concentration of antidepressants, it led to a decrease in growth rate comparable to the abovementioned antidepressants. Fluvoxamine had the least effect on growth activity. At a concentration of 200 μg/ml, it reduced the growth rate by 1.2 (17%) times compared with the control.

## 4. Discussion

The current study results confirm and expand the existing data on the antidepressant’s’ potential antimicrobial effects. In our experiment, we evaluated the *in vitro* effect of the most commonly prescribed antidepressants on commensal bacterial strains of the normal human gut microbiota. The results clearly showed that all antidepressants had an inhibitory effect on the growth of all studied microorganisms. All evaluated microorganisms play important role in the functioning of the host organism. Members of the *B. bifidum* species constitute one of the dominant taxa amongst the bifidobacterial communities and have been shown to display significant physiological and genetic aspects in encompassing adhesion to epithelia as well as the metabolism of host-derived glycans (Turroni et al., 2019). *S. aureus* is able to colonize multiple niches within humans, however the clinical implications of intestinal colonization by it are still relatively ill-defined (Sannasiddappa et al., 2011). The healthy gut microbiota including certain strains of *E. coli, E*. *faecalis*, and *L. rhamnosus* promotes intestinal homeostasis and can exert anti-inflammatory or even anti-cancer effects (Alhinai et al., 2019). The evolutionary advantage of *C. albicans* colonization may be the education of the host immune system and competition with other microorganisms with an impact on the balance of the intestinal microbiota (Kumamoto et al., 2020).

There are studies that show the association between a decrease in the taxonomic diversity of normal intestinal microflora representatives with depression (Simpson et al., 2021), which may be the reason for the decrease in the therapeutic effect with prolonged use of the drugs. On the other hand, experimental data suggest that the effect on the microbiota may be one of the mechanisms of antidepressant activity (O’Mahony et al., 2015). The direct effect of the intestinal microbiota on the serotonergic system is by limiting the availability of tryptophan for the host organism due to its need for the growth of bacterial strains and, subsequently, the synthesis of indole (Lee and Lee, 2010; Li and Young, 2013). Thus, it is possible that in certain cases, the antimicrobial activity of antibiotics can alter the balance of tryptophan, which will affect the level of serotonin and other neurotransmitters and, accordingly, the potential therapeutic effect.

Fluvoxamine and venlafaxine had the least effect on the growth activity of all studied microorganisms. These results are inconsistent with a previous experiment where venlafaxine at a maximum dosage of 600 μg/ml had no effect on the growth activity of both *E. coli* and *L. rhamnosus* (Ayaz et al., 2015; Ait Chait et al., 2020).

The effect of fluvoxamine on the growth activity of microorganisms was comparable to that of venlafaxine. Previous studies evaluating the antimicrobial activity of fluvoxamine and other SSRIs have shown that fluvoxamine has greater antibacterial activity against *E. coli* than *S. aureus* (McGovern et al., 2019). The results of these studies reported that the minimum inhibitory concentrations of fluvoxamine for *E. coli* and *S. aureus* were 32 and 4,000 mg/L, respectively. In general, this correlates with the results of our study, where fluvoxamine reduced the growth activity of *E. coli* and *S. aureus* by 4.1 and 1.2 times, respectively, at minimal concentrations.

Previous studies with venlafaxine have shown no *in vitro* antibacterial effect of venlafaxine against *L. rhamnosus* and *E. coli* (Cussotto et al., 2019; Ait Chait et al., 2020).

According to the results of our experiment, fluoxetine showed a pronounced effect on growth activity against *E. coli*, *E. feacalis*, *S. aureus*, and the least effect against *C. albicans*. In another study, fluoxetine showed the greatest activity against gram-positive bacteria, including *S. aureus*. In relation to gram-negative, it also showed a pronounced inhibitory, but to a lesser extent (Kalayci et al., 2015). Our results showed that fluoxetine had the greatest effect on the growth activity to *E. coli*, reducing the growth rate by 6.9 times at a concentration of 200 μg/ml compared with the control. The antifungal activity of fluoxetine shown by our study is confirmed by other researchers (Lass-Flörl et al., 2001; Costa Silva et al., 2017).

Escitalopram in our experiment had a greater effect on the growth rate of *E. coli*, *E. feacalis*, *B. bifidum*, *L. rhamnosus*, and *C. albicans*, which puts it among the leaders in terms of its effect on the growth activity of the microorganisms we studied. These results do not confirm the earlier studies, where escitalopram showed one of the lowest antibacterial activities. In a study by Hendricks et al. (2007), the value of the minimum inhibitory concentration of escitalopram for *E. feacalis* was >256 mg/L. And in a study by Kalayci et al. (2015), escitalopram showed no effect on the growth of *E. feacalis*, *E. coli*, *C. albicans*, and *S. aureus*. However, a study by Irish scientists confirms the antibacterial activity of escitalopram against *E. coli* (Cussotto et al., 2019).

Mirtazapine, according to the results of our experiment, showed the greatest activity against *L. rhamnosus* and *C. albicans*. With respect to *S. aureus*, mirtazapine had a pronounced effect on growth activity only at high concentrations. Studies of mirtazapine and duloxetine are not numerous; therefore, it is not possible to provide a comparative analysis of our results. The only study of mirtazapine found showed no antimicrobial effect against all microorganisms studied by us (Kalayci et al., 2015).

Our results showed the variability of the antibacterial activity of the tested antidepressants in relation to different types of microorganisms. These differences in microbial suppression may contribute to changes in gut microbial diversity, causing a shift in microbial communities towards dysbiosis and eubiosis. Presumably the basis of antibacterial action of antidepressants, in particular SSRIs, are their ability to inhibit the active excretion of the drug from the microorganism cell (efflux effect; Kaatz et al., 2003). The complete understanding of such mechanisms still remains obscure. However, antidepressants may influence microbial diversity through strong selection for mutant bacteria with increased AcrAB-TolC activity, an efflux pump that removes antibiotics from cells. Moreover, a new group of genes that contribute to cross-resistance between antidepressants and antibiotics, act by regulating efflux activity, underscoring overlapping mechanisms, was identified (Ou et al., 2022).

## 5. Limitations

The main limitation of our study was its *in vitro* methodology. In order to apply current results to clinical practice, it is necessary to understand whether antidepressants reach the gastrointestinal tract in sufficient concentration to exert antimicrobial effects. Since the pharmacokinetics of drugs are affected by many factors, such as dosage, solubility, absorption, distribution, etc. it is difficult to estimate the actual concentrations of orally administered drugs in the gastrointestinal tract. Moreover, the time of exposure and the cumulative effect of antidepressants in the colon may be a determining factor for increased antimicrobial activity, since antidepressants are taken daily for a prolonged period of time.

## 6. Conclusions

Our results confirm the effects of antidepressants on the growth activity of individual strains of representatives of the normal gut microbiota. The results were depending on the studied drug and the dosage evaluated in the experiment. Therefore, there is evidence to believe that antidepressants may affect the gut microbiota, which may possibly influence the therapeutic process. But this assumption can be confirmed or refuted only by conducting further clinical studies, including the analysis of the taxonomic diversity of the microbiome of patients with affective disorders. Further study of the antimicrobial activity of antidepressants on the intestinal microbiota may become one of the new directions for optimizing the personalized therapy of patients with depression, considering individual microbiome profiles.

## Data availability statement

The original contributions presented in the study are included in the article, further inquiries can be directed to the corresponding author.

## Author contributions

GM, NN, GR, and EK: study concept and supervision, manuscript revision and editing. LL and VL: methodology, experiment organization and conduction, statistical analysis. LL: text original draft. All authors contributed to the article and approved the submitted version.

## Footnotes

^1^ www.graphpad.com
